# Topical fluoride and regulation of salivary pH in Peruvian Altiplano schoolchildren: a comparative longitudinal study

**DOI:** 10.3389/froh.2025.1620432

**Published:** 2025-06-19

**Authors:** Vilma Mamani-Cori, Talia Paola Calcina-Asillo, Marleny Chino-Mamani, Yang Rodrigo Mendoza-Quispe, Sidgar Orlando Yucra-Sardón, Heber Isac Arbildo-Vega, Tania Carola Padilla-Cáceres, Betsy Quispe-Quispe, Franz Tito Coronel-Zubiate

**Affiliations:** ^1^ Department of General Dentistry, Dentistry School, University of the Altiplano, Puno, Peru; ^2^ Department of General Dentistry, Dentistry School, San Martín de Porres University, Chiclayo, Peru; ^3^ Department of Human Medicine, School of Human Medicine, San Martín de Porres University, Chiclayo, Peru; ^4^ Department of Health Sciences, Stomatologist School, Universidad Nacional Toribio Rodríguez de Mendoza de Amazonas, Chachapoyas, Peru

**Keywords:** cariostatic agents, gels, saliva, sodium fluoride, fluorides, topical

## Abstract

**Introduction:**

Saliva acts as a natural buffer, neutralizing the acids produced by bacterial metabolism. Maintaining salivary pH in a range close to neutrality is essential for enamel remineralization processes. This study aimed to evaluate and compare the effect of different concentrations and formulations of topical fluoride on the regulation of salivary pH in schoolchildren from the Altiplano region of Peru.

**Method:**

A quantitative, longitudinal, double-blind, randomized experimental design was employed. A total of 200 children aged 6–12 years who voluntarily agreed to participate were randomly and equally distributed into four study groups. To ensure homogeneous assignment, sociodemographic variables (sex, family type, age, number of siblings, and frequency of daily brushing) and clinical variables (caries severity and oral hygiene level) were controlled. Data were analyzed using SPSS version 25.0. The Shapiro–Wilk test assessed normality (*p* < 0.05), while Kruskal–Wallis test was used for between-group comparisons and the Friedman test for intragroup comparisons.

**Results:**

Intragroup analysis revealed statistically differences in salivary pH at initial, post-brushing, 10, 30, 60 min and 24-hours measurements across all groups (Friedman's Test; *p* < 0.001). Between-group comparisons also showed significant differences in salivary pH at 10, 30, 60 min, and at 24 and 48 h (Kruskal–Wallis test; *p* < 0.05).

**Conclusion:**

In conclusion, the 5% fluoride varnish and fluoride gels (1.23% and 2%) demonstrated greater efficacy in regulating salivary pH, especially during the initial hours following application.

## 1 Introduction

Dental caries is one of the most prevalent oral diseases worldwide, disproportionately affecting vulnerable populations such as children, individuals from disadvantaged socioeconomic backgrounds, and those living in rural or geographically isolated areas (1–3). The development of dental caries is multifactorial, involving a dynamical interaction between essential and environmental factors (2, 4–6). Among the essential factors, the presence of cariogenic microorganisms, the availability of fermentable substrates, and host characteristics are critical; notably, saliva plays a key role in maintaining oral homeostasis by acting as a protective barrier against enamel demineralization (7–10).

Saliva is composed predominantly of water (approximately 99%), along with electrolytes (such as calcium, phosphate, bicarbonate and fluoride), proteins, enzymes and antimicrobial molecules, which collectively contribute to the maintenance of oral equilibrium (11, 12). Regarding pH regulation, saliva functions as a buffering system that neutralizes acids produced by bacterial metabolic activity. This buffering capacity, mainly mediated by the bicarbonate system, maintains salivary pH within a near-neutral range, which is essential for enamel remineralization (11, 12). Additionally, saliva facilitates the transport and retention of fluoride ions, enhancing its remineralizing effects by increasing fluoride ions, enhancing its remineralizing effects by increasing fluoride availability at the tooth surface (12, 13).

Salivary pH not only reflects the acid-base balance of the oral environment but also serves as an indicator of cariogenic risk (11). A low pH, resulting from acid production by cariogenic bacteria such as *Streptococcus mutans* and *Lactobacillus* spp., favor enamel demineralization and, in the absence of protective mechanisms, can lead to irreversible carious lesions. In contrast, a neutral or slight alkaline pH promotes enamel remineralization, supported by the availability of calcium and phosphate ions in the saliva (11, 14).

Topical fluoride has been established as a key preventive agent against dental caries due to its physicochemical and biological properties (9, 15, 16). Beyond promoting the formation of fluorapatite, topical fluoride increases fluoride ion concentrations in the oral environment, enhances salivary buffering capacity, and slows the rate of pH decline following sugar ingestion (17). These actions not only inhibit bacterial metabolic activity—thereby reducing organic acid production—but also contribute to stabilizing the oral microenvironment by promoting a dynamic balance between demineralization and remineralization.

Understanding and addressing the factors that influence salivary pH and their relationship with caries progression is particularly critical in vulnerable populations, such as schoolchildren from the Peruvian Altiplano, who face distinct environmental and biological challenges. In this context, topical fluoride emerges as a fundamental tool, not only for its capacity to promote enamel remineralization but also for its interaction with saliva in stabilizing oral pH and mitigating the effects of metabolic acids. However, the comparative efficacy of different topical fluoride formulations in regulating salivary pH under these specific conditions remains insufficiently studied.

Therefore, the aim of this study was to evaluate and compare the effects of different concentrations and formulations of topical fluoride on the regulation of salivary pH in schoolchildren from the Peruvian Altiplano. This study seeks to generate scientific evidence to support caries prevention strategies tailored to the needs of this population and contribute to the development of effective and culturally contextualized oral health policies aimed at reducing oral health inequities.

## 2 Materials and methods

### 2.1 Study design and participants

This quantitative, longitudinal, double-blind, randomized experimental study was conducted between September and December 2024. Participants were recruited from three rural schools located in the province of Puno, Peru. Healthy children aged 6–12 years were included. Participation was voluntary, and informed consent was obtained from parents or legal guardians, along with the assent of the children.

Exclusion criteria included having received preventive fluoride treatments within the 3 months preceding the study, presenting advanced dental caries with pulp exposure or abscess formation, or exhibiting clinical signs of xerostomia, such as dry oral mucosa or an inability to produce sufficient saliva within 5 min during the initial screening.

The sample size was determined by a calculation based on a minimum expected difference of 0.3 pH units between groups, with a 95% confidence interval, 80% statistical power, and an estimated non-response rate of 10%. The result was a sample size of 50 participants per group, totaling 200 schoolchildren.

Assignment to the four groups was done through stratified randomization by age and sex, using a random numbers table generated in SPSS v25. An external researcher, not involved in the intervention or data collection, performed the allocation concealment.

The intervention groups were as follows:

Group 1: 1.23 acidulated sodium fluoride gel (Maquira®, Brazil).
Group 2: 2% neutral sodium fluoride gel (Maquira®, Brazil).
Group 3: 5% sodium fluoride varnish with tricalcium phosphate (3 M™ Clinpro™ White Varnish, USA).
Group 4: Fluoride toothpaste with 1,450 ppm sodium monofluorophosphate (Kolynos®, Colgate-Palmolive Company, Andean Region).

**Figure 1:**
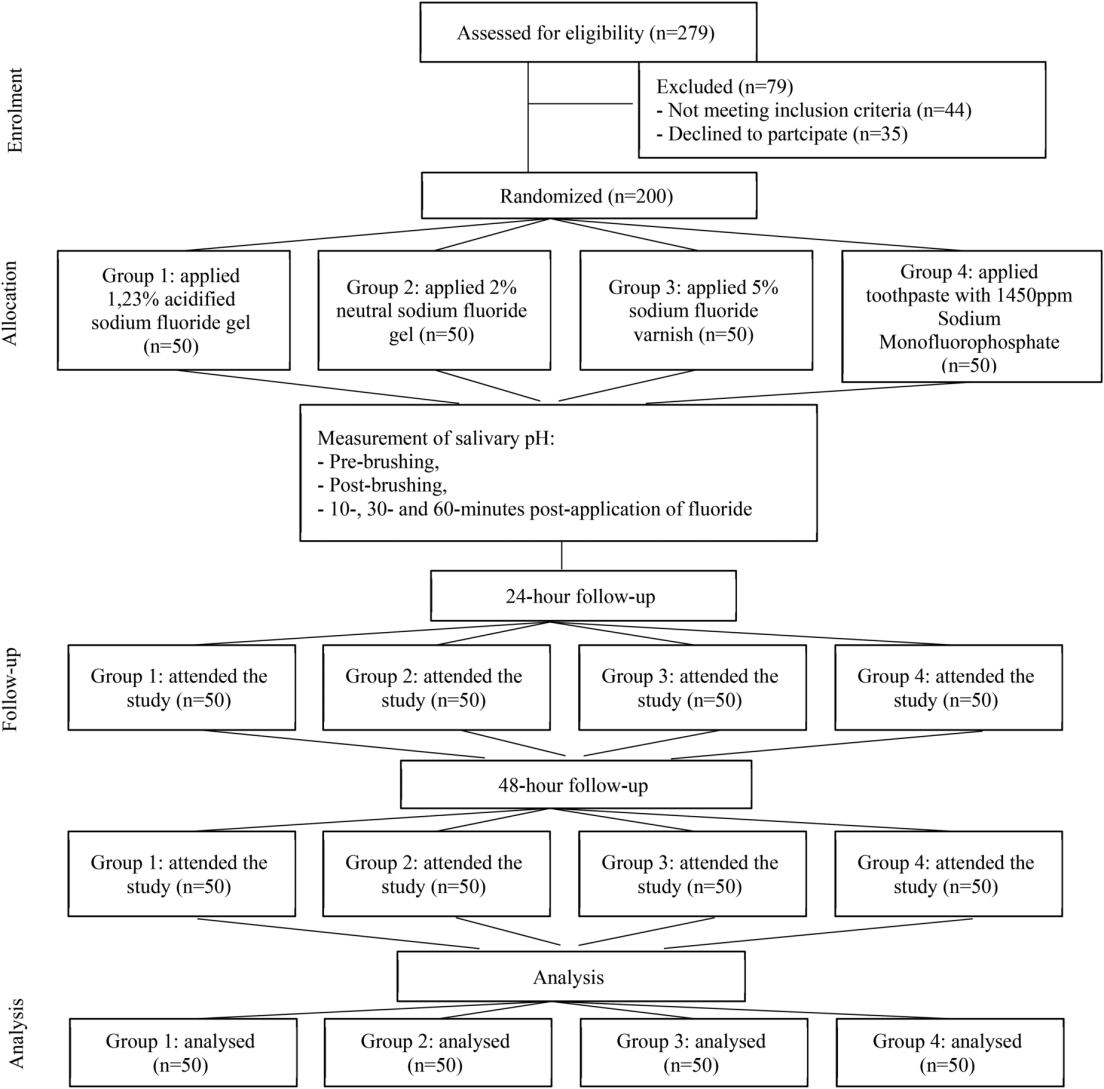
CONSORT flow diagram illustrating the enrollment, allocation, follow-up, and analysis of participants in the study.

The group allocation and intervention details are summarized in Figure 1 (CONSORT flow diagram).

### 2.2 Data collection

Data collection was performed under double-blind conditions. Five examiners participated: four calibrated examiners (inter-examiner kappa = 0.81) evaluated sociodemographic and clinical variables and applied the topical fluoride treatments according to manufacturers’ instructions. A fifth independent examiner, blinded to group assignment, measured salivary pH using Hanna® digital pH meter (accuracy ± 0.2 pH units). Data were collected at an average ambient temperature of 15.8°C and an average humidity of 54%, between 9 a.m. and 12 p.m.

### 2.3 Homogenization of study groups

Prior to intervention, participants were stratified based on sociodemographic and clinical characteristics.

Sociodemographic data (sex, family type, age, number of siblings, and frequency of daily tooth brushing) were collected using a structured questionnaire. The questionnaire was developed by the research team based on prior validated instruments used in oral health studies. Content validity was assessed by a panel of three experts in pediatric dentistry and public health, who evaluated item relevance and clarity. A pilot test was conducted with 20 children not included in the main study to ensure comprehensibility and refine the final version.

Clinical assessment included caries severity, evaluated with the International Caries Detection and Assessment System (ICDAS) (18), and oral hygiene, assessed using the Simplified Oral Hygiene Index (OHI-S) of Green and Vermillion (19). This ensured homogeneity across groups and minimized confounding variables.

### 2.4 Calibration of measuring equipment

The pH meter was calibrated using standard buffer solutions (pH 4.01, 6.86, and 9.18) prepared with sterile water, following the manufacturer's guidelines. Calibration was verified every 10 measurements to maintain the precision.

### 2.5 Saliva collection

Unstimulated whole saliva specimens were collected under standardized conditions. Participants refrained from eating, drinking (except water), tooth brushing, or chewing gum for at least 1 h before collection.

Participants were instructed to expectorate a-5 ml of saliva into sterile containers within a maximum of 5 min. Specimens were labeled and coded before salivary pH measurement.

Collection was performed at following times:

• Pre-brushing (baseline).
• Post-brushing (after fluoride toothpaste).
• Post-fluoride application (10, 30, 60 min, 24 and 48 h).

Infection control protocols were strictly followed during saliva collection. Examiners wore personal protective equipment (PPE), including gloves, masks, and disposable gowns. Work surfaces were disinfected before and after each session, and all saliva containers and collection instruments were sterile and single-use.

### 2.6 Salivary pH measurement

Salivary pH was assessed immediately after collection using the calibrated Hanna® digital pH meter. Measurements were recorded at each time point to monitor the temporal changes associated with fluoride treatments.

### 2.7 Tooth brushing protocol

Following the collection of the baseline saliva sample, participants brushed their teeth using fluoride toothpaste (1,450 ppm sodium monofluorophosphate) with the “sweeping technique”. A pea-sized amount (equivalent to a lentil) was used for children aged 6–9 years, and one-third of a toothbrush head for children aged 10–12 years.

### 2.8 Intervention

Topical fluoride was applied according to the group assignment:

• Group 1: 1.23% acidulated sodium fluoride gel.
• Group 2: 2% neutral sodium fluoride gel.
• Group 3: 5% sodium fluoride varnish with tricalcium phosphate.
• Group 4: Fluoride toothpaste (1,450 ppm sodium monofluorophosphate).

Manufacturers’ application instructions were strictly followed.

Participants were instructed to avoid eating or drinking during the immediate post-application measurement intervals (10, 30 and 60 min).

### 2.9 Follow-up

Participants were followed for 48 h after fluoride application. Salivary pH samples were collected at 24 and 48 h, under the same fasting conditions as baseline collections.

### 2.10 Ethical considerations

The study was approved by the Ethics Committee of the Universidad Nacional del Altiplano (Approval Certificate No. 065/CIEI UNA-Puno; Registration Code 103-CIEI UNA Puno).

The research adhered to the ethical principles of the Declaration of Helsinki (20) and complied with Peru's Personal Data Protection Law (Law No. 29733), ensuring participant confidentiality and non-discrimination. Informed consent and child assent obtained from all participants and their legal guardians.

### 2.11 Statistical analysis

All data were entered into a digital database and analyzed using SPSS version 25.0 (IBM Corp., Armonk, NY, USA). Salivary pH data were first assessed for normality using Shapiro–Wilk test (*p* < 0.05). Give the non-normal distribution, nonparametric tests were applied: the Kruskal–Wallis test for comparisons between groups and the Friedman test for comparisons within groups. Statistical significance was set at *p* < 0.05.

## 3 Results

A total of 200 schoolchildren from the Peruvian Altiplano participated in the study and were evenly distributed into four study groups. Table 1 presents the descriptive analysis of the sociodemographic and clinical characteristics, confirming the homogeneity of the groups prior to intervention.

**Table 1:** Descriptive analysis of sociodemographic and clinical characteristics of the participants.

Characteristics	Fluoride gel 1.23%	Fluoride gel 2%	Fluoride varnish 5%	Fluoride toothpaste (1,450 ppm)	*p*-value*
Sex					0.352
Male	27 (54.0%)	23 (46.0%)	18 (36.0%)	23 (46.0%)	
Female	23 (46.0%)	27 (54.0%)	32 (64.0%)	27 (54.0%)	
Family type					0.149
Nuclear family	44 (88.0%)	35 (70.0%)	40 (80.0%)	37 (74.0%)	
Single-parent family	6 (12.0%)	15 (30.0%)	10 (20.0%)	13 (26.0%)	
Age (mean ± SD)	9.3 ± 1.9 (6–12)	9.8 ± 1.2 (6–12)	9.5 ± 1.6 (6–12)	9.6 ± 1.7 (6–12)	0.493
Number of siblings (mean ± SD)	1.2 ± 0.8 (0–3)	1.2 ± 1.2 (0–6)	1.1 ± 1.1 (0–5)	1.2 ± 0.9 (0–4)	0.588
Frequency of daily brushing					0.337
No brushing	1 (2.0%)	1 (2.0%)	2 (4.0%)	1 (2.0%)	
Less than once daily	16 (32.0%)	14 (28.0%)	7 (14.0%)	14 (28.0%)	
Once daily	21 (42.0%)	31 (62.0%)	30 (60.0%)	26 (52.0%)	
Twice or more daily	12 (24.0%)	4 (8.0%)	11 (22.0%)	9 (18.0%)	
Severity of caries					0.139
Mild	6 (12.0%)	10 (20.0%)	6 (12.0%)	7 (14.0%)	
Moderate	15 (30.0%)	22 (44.0%)	18 (36.0%)	16 (32.0%)	
Severe without pulp involvement	29 (58.0%)	18 (36.0%)	26 (52.0%)	27 (54.0%)	
Oral hygiene					0.175
Regular	22 (44.0%)	24 (48.0%)	27 (54.0%)	17 (34.0%)	
Good	26 (52.0%)	20 (40.0%)	22 (44.0%)	28 (56.0%)	
Excellent	2 (4.0%)	6 (12.0%)	1 (2.0%)	5 (10.0%)	
Total	50 (100.0%)	50 (100.0%)	50 (100.0%)	50 (100.0%)	

* Kruskal–Wallis Test.

Regarding sex distribution, no significant differences were observed between groups (*p* = 0.352), with similar proportions of males and females. The predominant family type was nuclear, ranging from 70% to 88% across groups (*p* = 0.149). The mean age of participants ranged between 9.5 and 9.8 years (*p* = 0.493), and the average number of siblings varied from 1.1 to 1.2 (*p* = 0.588), with no significant intergroup differences.

Most participants reported brushing their teeth once daily, with brushing two or more times being slightly frequent in the fluoride varnish group (22%) and less common in the 2% fluoride gel group (8%), though differences were not statistically significant (*p* = 0.337). Regarding caries severity, severe cases without pulp involvement predominated, particularly in the 1.23% fluoride gel (58%) and fluoride varnish (52%) groups (*p* = 0.139). In terms of oral hygiene, higher proportions of good and excellent levels were observed in the varnish (60%) and 2% fluoride gel (56%) groups (*p* = 0.175).

Overall, the data show the homogeneity of the groups, ensuring the comparability of outcomes post-intervention.

Table 2 presents the comparative analysis of the effect of different topical fluoride treatments on salivary pH regulation across various time points: baseline, post-brushing, and 10, 30, 60 min, 24, and 48 h post-application.

**Table 2:** Comparative analysis of the effect of topical fluoride treatments on saliva pH.

Salival pH Groups		Initial	Post-brushing	Post-fluoridation			Post-brushing		*p*-value
				10 min	30 min	60 min	24 h	48 h	
Fluoride gel 1.23%	Mean	6.82	7.01	6.95	7.02	7.11	7.08	7.04	<0.001*
	SD	0.27	0.33	0.4	0.17	0.13	0.18	0.2	
	Min.	6.08	5.12	4.86	6.42	6.69	6.23	6.02	
	Max.	7.69	7.46	8.1	7.45	7.42	7.38	7.45	
Fluoride gel 2%	Mean	6.89	7.12	7.12	7.14	7.15	7.1	7.06	<0.001*
	SD	0.29	0.26	0.22	0.2	0.3	0.15	0.17	
	Min.	6.05	6.6	6.64	6.65	5.75	6.75	6.63	
	Max.	7.63	8.13	7.63	7.65	7.79	7.45	7.40	
Fluoride varnish 5%	Mean	6.85	7.06	7.15	7.18	7.24	7.16	7.12	<0.001*
	SD	0.28	0.24	0.2	0.21	0.33	0.13	0.13	
	Min.	5.56	6.14	6.76	6.66	6.83	6.79	6.81	
	Max.	7.7	7.55	7.65	7.82	8.6	7.45	7.38	
Fluoride toothpaste 1,450 ppm	Mean	6.81	7.04	7.07	7.08	7.06	7.05	7.05	<0.001*
	SD	0.22	0.25	0.27	0.22	0.29	0.17	0.18	
	Min.	6.2	6.15	6.26	6.5	5.59	6.6	6.57	
	Max.	7.21	7.5	7.96	7.43	7.47	7.42	7.45	
	*p*	0.446**	0.289**	<0.001**	0.001**	0.026**	0.003**	0.149**	

* Test de Friedman.

** Kruskal–Wallis.

In general, all interventions led to an increase in salivary pH compared to baseline, with progressive stabilization by 48 h.

For the 1.23% fluoride gel, the mean baseline pH was 6.82, slightly increasing to 6.95 at 10 min, and reaching 7.08 at 24 h, with stabilization at 7.04 by 48 h.

The 2% fluoride gel showed an initial pH of 6.89, rising to 7.12 at 10 min, and maintaining a value of 7.06 at 48 h, indicating a relatively homogeneous pH regulation pattern.

The 5% fluoride varnish demonstrated a baseline pH of 6.85, with a sustained increase to 7.24 at 60 min, and a slight decrease to 7.12 at 48 h. This group maintained the highest pH levels during the first 60 min compared to the other interventions.

The fluoride toothpaste (1,450 ppm) presented a baseline pH of 6.81, achieving stabilization around 7.08 at 30 min, and a slight decrease to 7.05 at 48 h. Although positive, the magnitude of pH regulation was lower compared to the other treatments.

Intragroup comparison using the Friedman test showed statistically significant differences in salivary pH at all time points for each study group (*p* < 0.001). Intergroup Comparisons using the Kruskal–Wallis test revealed significant differences in salivary pH at 10, 30, 60 min and 24 h post-application (*p* < 0.05), but no significant differences were found at baseline, post-brushing, or 48 h (*p* > 0.05).

This suggests a homogeneous starting point and final stabilization across treatments.

**Figure 2:**
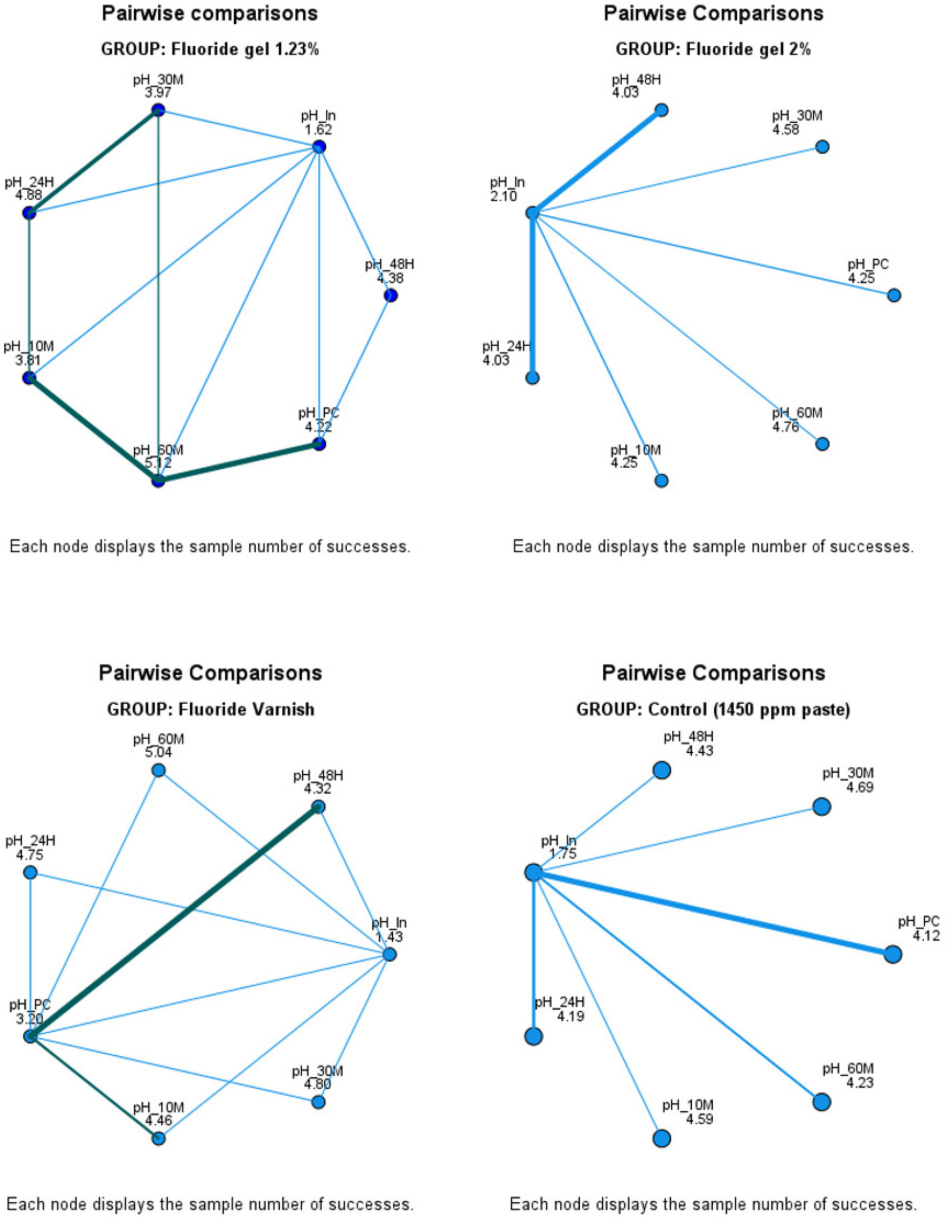
The results illustrate that the fluoride varnish demonstrated the most sustained effect on salivary pH alkalinization, achieving high levels at 24 and 48 h post-application. This suggests its superior capacity for prolonged fluoride release. The 1.23% fluoride gel also promoted a significant pH increase, though with a shorter persistence compared to the varnish. In contrast, the 2% fluoride gel exhibited a rapid alkalinizing effect, but with lower durability over time. Finally, the group using fluoride paste with 1,450 ppm showed more limited pH changes and earlier stabilization. These findings underscore the importance of selecting the appropriate fluoride treatment according to specific needs, particularly in at-risk populations such as schoolchildren from Altiplano region.

The salivary pH values compared at different time points after the application of various fluoride treatments. Figure 2 illustrates the dynamic changes observed across the groups.

These results support the hypothesis that fluoride varnish formulations provide a more sustained protective effect against acidogenic challenges in the oral cavity. Moreover, they highlight the need for individualized preventive strategies depending on the patient's risk profile and environmental factors.

## 4 Discussion

This study evaluated the effects of different concentration and presentation of topical fluoride on salivary pH regulation in schoolchildren from the Peruvian Altiplano.

The results showed that all interventions increased salivary pH compared to baseline values, with a progressive stabilization at 48 h. Initially, mean salivary pH values across the four groups ranged from 6.81 (±0.22) to 6.89 (±0.29), consistent with values reported by Gonzáles-Aragón et al. (6.9 ± 0.54) and Pyati et al. (6.64 ± 0.54) (11, 12).

This research confirmed that all topical fluoride interventions increased salivary pH, with notable differences in magnitude and duration. The sustained alkalinizing effect of the 5% fluoride varnish aligns with findings from Soares-Yoshikawa et al., Senthilkumar et al. and Casimiro-Iriarte et al., who reported prolonged fluoride release and caries-preventive effects from varnish formulations (21–23).

Conversely, the moderate persistence observed with 1.23% and 2% fluoride gels is consistent with earlier trials indicating a shorter duration of fluoride availability in gel-based applications. This observation is supported by findings from Turska-Szybka et al. (24) y Ribeiro et al. (25) who reported that salivary fluoride concentrations rose sharply after gel application but decreased significantly within 60 min. This reinforces the notion that gel formulations exhibit rapid clearance from the oral cavity and shorter salivary retention compared to varnishes (26, 27).

Interestingly, the rapid but less durable alkalinization seen with 2% neutral fluoride gel may be clinically useful for short-term pH regulation, a hypothesis also raised by Polyakova et al. (14). The limited effect observed with the 1,450 ppm fluoride toothpaste parallels previous work by Cagetti et al. (16), highlighting that standard toothpaste formulations may offer only transient buffering benefits, particularly in populations with high cariogenic risk.

These findings highlight the importance of selecting the appropriate fluoride intervention based on specific population needs. In the context of schoolchildren from the Peruvian Altiplano-who may be exposed to environmental and dietary factors that heighten their risk of dental caries-the use of fluoride varnish could be particularly advantageous due to its extended protective effects.

Despite the methodological strengths of this study, including a controlled experimental design and examiner calibration, certain limitations must be acknowledged. First, although efforts were made to minimize interindividual variability in saliva collection for pH measurement, it is recognized that the schoolchildren's diet was not controlled during the collection period, which could influence salivary pH values. Second, the follow-up was limited to 48 h, which may not fully capture long-term pH modulation. Third, dietary intake during the follow-up period was not standardized, potentially influencing salivary pH. Lastly, fluoride uptake at the enamel level was not measured, which could have provided further insight into clinical effectiveness.

Future research should incorporate extended follow-up, control for dietary variables, and consider biochemical measures of fluoride uptake to better understand the long-term effects of different topical fluoride formulations in high-altitude pediatric populations.

## 5 Conclusion

The 5% fluoride varnish demonstrated the most sustained efficacy in regulating salivary pH, maintaining elevated levels up to 48 h post-application. The 1.23% and 2% fluoride gels also increased salivary pH significantly, although with less persistence over time. These findings underscore the importance of selecting topical fluoride treatments based on their release profiles and duration effect. In population such as schoolchildren from high-altitude regions, where environmental and dietary factors may heighten caries risk, high-concentration fluoride products-particularly varnishes-appear especially beneficial for strengthening preventive dental strategies adapted to local conditions.

## Data Availability

The raw data supporting the conclusions of this article will be made available by the authors, without undue reservation.
